# Case Report: Systemic lupus erythematous associated with thrombotic thrombocytopenic purpura, a diagnostic challenge

**DOI:** 10.12688/f1000research.51295.2

**Published:** 2022-05-25

**Authors:** Horacio Suárez-Ale, Elizabeth Fabian-Aquino, Virgilio E. Failoc-Rojas, Vicente A Benites-Zapata, Felipe Ignacio-Cconchoy

**Affiliations:** 1 Internal Medicine Service, Hospital Nacional Alberto Saboga Sologuren, Lima, Peru; 2 Nefrology Service, Hospital Nacional Alberto Sabogal Sologuren, Lima, Peru; 3 Unidad de Investigación para la Generación y Sintesis de Evidencias en Salud, Universidad San Ignacio de Loyola, Lima, Peru

**Keywords:** Systemic Lupus Erythematous, Thrombotic Thrombocytopenic Purpura, plasmapheresis, adamts-3 protein, human

## Abstract

Thrombotic thrombocytopenic purpura (TTP) is an uncommon microangiopathic disease and sometimes is associated with systemic lupus erythematous (SLE). However, this probable causal relationship has not been completely proven. The diagnostic differentiation of both diseases is difficult in the first instance because they share similar characteristics that may overlap. We present a case of a 32-year-old woman with antecedents of epileptic seizures since she was 12 years old. The patient was admitted to the emergency room with a clinical picture of headaches, fever, paleness in the skin and mucosa, confused state, paresthesia, and transient spasticity of the extremities. The laboratory results revealed direct Coombs negative hemolytic anemia, severe thrombocytopenia, significant elevation of lactate dehydrogenase, and presence of schistocytes ++ in the peripheral film. In addition, positive antinuclear antibodies and positive anti-native DNA in titers of 1/320 and 1/160, respectively, were found. Urinalysis showed that serum creatinine was in normal range. Because of limited hospital resources, ADAMTS13 was not evaluated. However, based on clinical, hematological, and biochemical findings, we concluded that it was a case of TTP associated with SLE and indicated treatment with plasmapheresis and methylprednisolone pulses, obtaining a satisfactory response (normalization of biomarker levels, health condition) after the second session of plasmapheresis. Diagnosis of both SLE and TTP is often difficult to achieve; however, adequate correlation of clinical manifestations and laboratory tests, along with the help of partial therapeutic interventions, may lead to good clinical response.

## Introduction

Thrombotic thrombocytopenic purpura (TTP) is a thrombotic microangiopathy (TMA) that can be classified as idiopathic or in association with other pathological processes such as neoplasias, infections, and autoimmune diseases such as systemic lupus erythematous (SLE). ^1^ Its classical presentation and characteristics are the pentad composed of fever, neurological disorder, renal dysfunction, microangiopathic hemolytic anemia, and thrombocytopenia. ^1, 2^ However, most of its presentation occur solely on some of these criteria, which makes diagnosis difficult. The occurrence of TTP associated with SLE has an immunological basis related to the formation of antibodies that inhibit or diminish the function of a disintegrin-like and metalloprotease with thrombospondin type 1 motif no. 13 (ADAMTS13), and the increase in serum level of the inhibitor of metalloprotease ADAMTS3. The presentation of both entities is rare, but if it occurs, TTP seems to be a complication of SLE. ^2, 3^

The clinical picture of both entities is somewhat similar because they share clinical characteristics that can overlap. It is difficult to make the diagnostic differentiation in the first instance, which delays the decision with regards to the correct treatment. This is important because the prognosis is more ominous when both entities appear at the same time, either as an association, or a secondary complication. We present a clinical case of a woman with a clinical presentation of SLE and TTP that was difficult to diagnose.

## Case presentation

We report a case of a 32-year-old mestizo woman with no specific occupation who was admitted to the hospital as an emergency because she was presenting with a confused state, paresthesia, and transient spasticity of the extremities that lasted for 30 minutes. She also reported she had a fever of 38°C for three days before admission. As important antecedents she reported headaches, dizziness, sporadic ecchymosis on the legs, and oral ulcers since she was a teenager, which she did not consider important. She has suffered from epileptic seizures since the age of 12 and has been treated inconsistently with carbamazepine. She also has an obstetric antecedent of three preterm labors between 32 and 35 weeks. The births were live and healthy and there were no pregnancy losses. Among family antecedents, her mother is epileptic, the grandmother on her mother’s side has lupus, and her youngest daughter had convulsions at three months of age.

**Figure 1.**
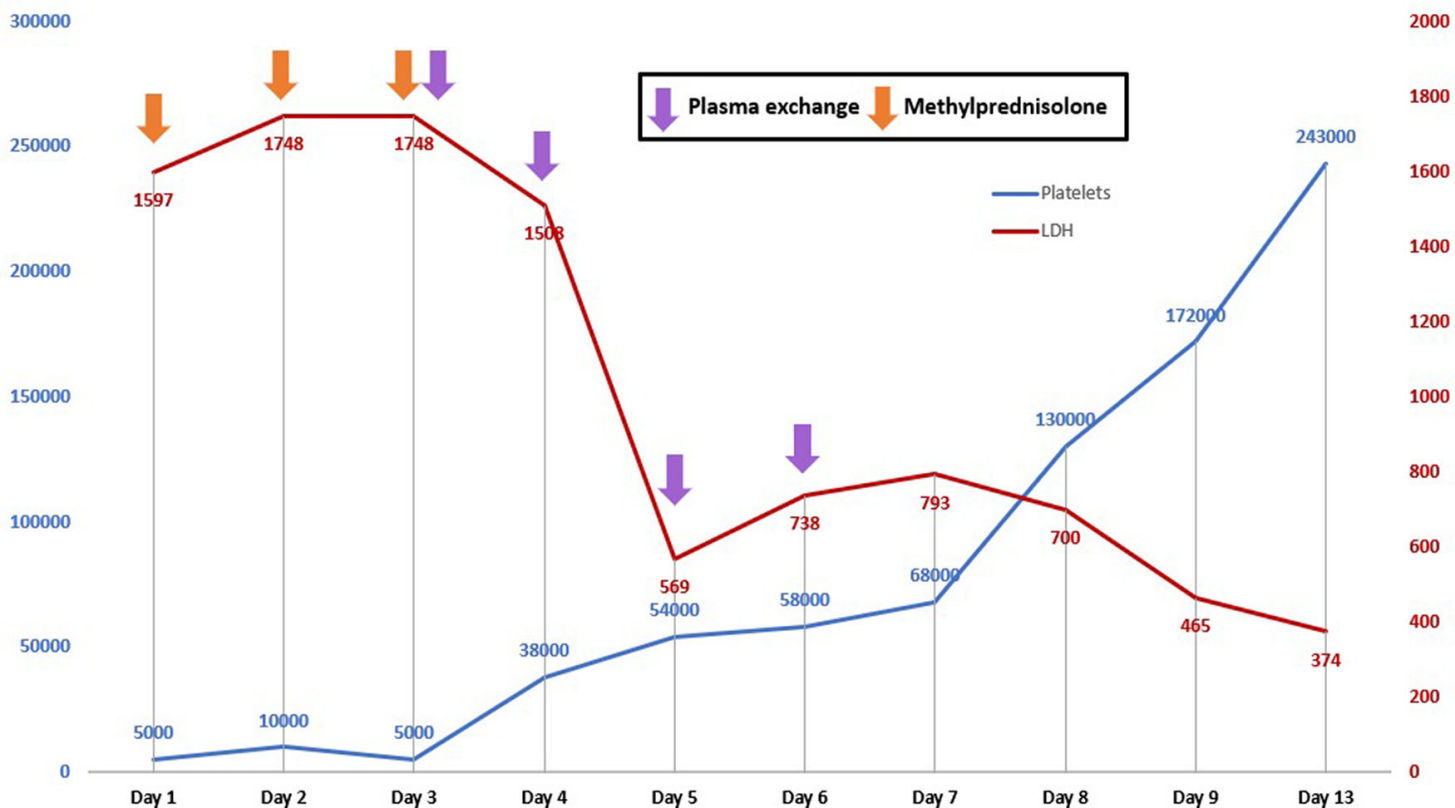
Evolution of platelets and LDH with established treatment in a patient with systemic lupus erythematous and thrombotic thrombocytopenic purpura. LDH: Lactate dehydrogenase.

On physical examination, a confused state, slight malar erythema, and ecchymosis on the legs and arms were found. The osteomuscular articular system and the cardiovascular and pulmonary systems had no alterations, and she was hemodynamically stable. On admission, blood test showed hemoglobin: 7.5 g/dL, reticulocytes: 11.92%, and platelet count: 10,000/mm ^3^; biochemical tests showed lactate dehydrogenase (LDH): 1,741 U/L, total bilirubin: 0.9 mg/dL, C-reactive protein (CRP) level: 0.79 mg/L, globular sedimentation rate (GSR): 77 mg/L, urea: 16 mg/dL (normal range: 11-20 mg/dL), and creatinine: 0.9 mg/dL (normal range: 0.7-1.5 mg/dL). Peripheral blood smear showed positive schistocytes (++). Brain CT-scan showed no alterations. Because of the clinical, hematological, and biochemical alterations reported, 300 mL of platelet concentrates were transfused five times, elevating the platelet count to 40,000/mm ^3^ with a rapid decline on the second day. This made us suspect that it was a case of thrombotic microangiopathy (TMA) with hemolytic anemia. We indicated autoantibodies tests in which we found negative antiphospholipid antibodies, positive antinuclear antibodies in titers of 1/320, and positive anti-native DNA in titers of 38.24 IU/mL. On the third day after admission, the platelets decreased to 5,000/mm ^3^ and the patient presented with a sudden neurological disorder with language disturbance, paresthesia in lower extremities, and confused state, totally recovering in few hours; an emergency brain CT-scan was performed, which showed no significant changes. Based on the history of epileptic seizures, oral ulcer and malar erythema at admission, plus subsequent immunological tests, the diagnosis of SLE was confirmed. In addition, TTP was diagnosed based on the two positive results of the systemic autoantibodies, along with severe thrombocytopenia, microangiopathic hemolysis, and the neurological disorder. We began treatment with methylprednisolone pulses of one gram IV every 24 hours for three days; however, three days after the beginning of treatment, the level of platelets and red blood cells decreased to 7,000 mm ^3^ and 6.8 g/dL, respectively, so we decided to begin plasma exchanges (plasmapheresis) using fresh frozen plasma with a volume of 2,000 mL. After the first plasma exchange, a significant increase in the level of platelets was evidenced, together with the reduction of LDH; so, four exchanges were completed, obtaining, five days after the last exchange, a normalization of the platelets and LDH levels: 243,000/mm ^3^ and 374 U/L, respectively ( Figure 1). Hemoglobin levels also began to raise to 9.5 g/dL, and there was a normalization of total bilirubin levels (0.28 mg/dL) and CRP (0.18 mg/dL). The patient had no clinical evidence of neurological symptoms, with platelets: 277,000/mm ^3^ (normal range: 150,000-400,000/mm ^3^) and hemoglobin: 12.8 g/dL (normal range: 11.9-13.5 g/dL). She was discharged four weeks after hospitalization with a medical prescription of prednisolone at a dose of 0.5 mg/kg/day PO for four weeks and her usual anticonvulsants (valproic acid: 30 mg/kg/day and carbamazepine: 15 mg/kg/day). There were no significant adverse effects.

Thirteen months after discharge, the patient was in good health, became pregnant, and had a satisfactory delivery with normal levels of platelets and hemoglobin.

## Discussion

TTP occurs in about 2% of patients with SLE. ^2, 3^ TTP manifestation after SLE has been more frequent than both appearing simultaneously. ^4^ This case report is about a young woman who appears for the first time with SLE associated with TTP.

TTP shares clinical and laboratory characteristics with SLE, which makes the diagnosis and proper treatment difficult and delayed, ^5^ with a resulting elevation of mortality. Mortality due to TTP is greater than 80%, but when the appropriate treatment is followed with plasma exchange, the survival rate is greater than 80%. On the other hand, when TTP is present in patients with SLE, the episode is usually severe and lethal with a mortality rate of 34.1-62.5%. ^6– 8^ In this case report, the diagnosis was made by anamnesis and laboratory tests; the patient achieved clinical improvement and survived. The classical clinical pentad in the presentation of TTP: microangiopathic hemolytic anemia (MHA), thrombocytopenia, neurological disorder, renal dysfunction, and fever was not observed in this case; this has been observed previously, but it is not frequent to find this pentad currently. ^7, 8^ In most cases, TTP presents alone, without other diseases, but when it is associated or concomitant with other pathologies, whether autoimmune, neoplastic or infectious, its diagnosis is often challenging. Sometimes, the treatment of concomitant pathologies in TTP can mitigate or mask some components of the classical clinical pentad. In other cases, the poor specificity of the initial clinical manifestations of the TTP, such as the neurological deficit associated with ischemia (headaches, consciousness disorders), fatigue, and abdominal pain associated with hemorrhage delays appropriate treatment. ^5, 8^

A cohort study that evaluated the clinical characteristics and prognostic factors of 105 cases of TTP associated with SLE found that the occurrence of the neurological disorder alone or accompanied by renal disorder was significantly higher in the group of patients that died than in the ones that survived, suggesting that the neurological and renal deficits could be a possible risk factor for mortality in patients with TTP and SLE. ^7^ In this clinical case, the patient presented with sensory disorder and a confused state but recovered immediately and the analysis of images did not show cerebral lesions. The absence of kidney involvement in our patient could be because the clinical presentation of TTP was before or simultaneous to one of SLE, a presentation described as the least frequent in all cases reported to date. ^8, 9^ The appropriate treatment with plasma exchange and adequate hydroelectrolytic management probably prevented kidney involvement. This is an interesting situation because in most cases in which SLE appeared first, there was kidney involvement accompanying other clinical manifestations of TTP. This could explain the relative resistance to indicated treatments including plasma exchange in the cases reported, which showed a high mortality rate. ^6^

Several studies have found that treatment with glucocorticoids and plasma exchange can achieve remission of 65.7% in patients with SLE and TTP ^7, 8^ and it has been observed that some treatment options such as rituximab are used for refractory cases, achieving a good prognosis. ^3, 7, 8^ In this case, the initial treatment was followed with methylprednisolone pulses of 1 g/day because it was focused mainly on active SLE. However, the pulses were not favorable because after two consecutive days the clinical picture, mainly the neurological one, persisted, and anemia and thrombocytopenia were worse. Therefore, plasma exchange was performed with the aim of treating TTP. With this, the platelet level increased significantly and there was a decrease in serum LDH levels. This strongly suggests that management with plasma exchange should be a priority once TTP is suspected. Regardless of any concomitant disease, it is known that for every day of delay in the treatment of TTP, the risk of death increases. ^10^ However, due to diagnostic uncertainty and limited hospital resources, plasma exchange is often not available at the time and other treatment options must be adopted.

The clinical and hematological evolution of the present case, which was favorable and rapid, contrasts with most of the cases reported in which there was a delay in the response and a high mortality rate despite the use of methylprednisolone pulses, plasma exchange, and other options such as the use of rituximab. ^3, 7, 8^ The explanation to this particular situation could be that, excluding the positive serology in this patient (that is, the ANA and anti-native DNA antibody), the presentation of the clinical characteristics was not compatible with SLE but mainly with TTP, which could lead us to assume that SLE, for the moment, would be only serological and that its clinical manifestations could become clear in the future.

Testing for ADAMTS13 activity is a critical step in the diagnosis of TTP, but many hospitals in Peru, such as the one in the present report, lack this test due to the high cost. Despite this resource limitation, plasma exchange is recommended in the presence of a clear suspicion of TTP, regardless of ADAMTS13 testing and the presence of other pathologies such as SLE. This treatment should be continued until the first platelet counts appear within normal limits for two consecutive days. ^11^ However, in this report the plasma exchange treatment was momentarily discontinued after four days even though the platelet level was not in the normal range. This approach was applied because, according to the consensus of experts in our hospital, the patient showed a favorable laboratory pattern in which the platelet level increased and the LDH concentration decreased. After seeing consistent clinical improvement, it was finally decided not to restart the plasma exchange. Additionally, TTP should always be considered in patients with hemolytic anemia and thrombocytopenia, and especially in young women, in whom SLE and TTP may occur simultaneously.

The strength of this case report relies on the uncommon presentation of both SLE and TTP, which adds more information about the clinical picture and possible outcomes. The main limitation was the lack of ADAMTS13 activity and inhibitor tests, which are not routinely used in our setting due to limited resources, but could have helped to confirm the diagnosis of TTP. The diagnosis of both conditions was even more challenging considering the differential diagnosis of epilepsy and the inconsistent compliance with medication for this neurological disorder.

## Conclusion

SLE and TTP can occur together, although not frequently, and this makes diagnosis difficult. However, considering the serious consequences of not treating TTP (i.e., death or major organ damage), patients should be promptly treated as TTP with plasma exchange and steroids while the diagnosis is being resolved. As in our case, plasma exchange and steroids can be used successfully, and patients can achieve remission with treatment.

## Data availability

All data underlying the results are available as part of the article and no additional source data are required.

## Consent

Written informed consent was obtained from the patient for publication of this case report and any accompanying images.
