# Supplementary material for: Case Report: Systemic lupus erythematous associated with thrombotic thrombocytopenic purpura, a diagnostic challenge
Source: F1000Res. 2022 May 25;10:552. Originally published 2021 Jul 9. [Version 2] doi: 10.12688/f1000research.51295.2 (PMC10338979; doi:10.12688/f1000research.51295.2)
Supplement: Peer review history

# Peer review: Reviewer response for version 2

**DOI:** 10.5256/f1000research.134308.r179420

**Published:** 2023-07-12

**Authors:** Edinson Dante Meregildo-Rodriguez

**Affiliations:** 1 Escuela de Medicina, Cesar Vallejo University, Trujillo, La Libertad, Peru

**Title and Abstract**.

- Since ADAMTS13 activity and inhibitor testing were not performed, I suggest modifying the title and the entire manuscript to “Case Report: Systemic lupus erythematous associated with probable immune thrombotic thrombocytopenic purpura, a diagnostic challenge”.

**Introduction:**

- What’s the aim of this case presentation?

**Case presentation. **

- Although it is a rare phenomenon, did the clinician consider the possibility of a carbamazepine-induced SLE? The authors should mention the result of anti-histone antibodies if it was performed.
- The authors describe that at least 2 brain CT-scan were performed. However, they do not specify if these exams were contrasts enhanced or not. This question is relevant because TTP can induce cloth formation in the brain and vessels.
- In addition, TTP was diagnosed based on the two positive results of the systemic autoantibodies along with severe thrombocytopenia, microangiopathic hemolysis, and neurological disorder. This statement is inaccurate because the result of autoantibodies does not confirm the diagnosis of TTP.
- The authors do not mention Mean corpuscular volume (MCV) and International normalized ratio (INR). Both results are essential to calculate PLASMIC score.

**Discussion.**

- Given the limitations in establishing a definitive diagnosis, the authors should discuss the role of the PLASMIC score. The PLASMIC score can be easily calculated using findings available on presentation and has been devised to predict the likelihood of ADAMTS13 activity ≤10 percent and the probability (low, intermediate, or high) of TTP. Several studies and a systematic review confirmed the diagnostic accuracy of the PLASMIC score in individuals with suspected TTP ^1^^,^^2^.

Are enough details provided of any physical examination and diagnostic tests, treatment given and outcomes?

No

Is the case presented with sufficient detail to be useful for other practitioners?

Partly

Is sufficient discussion included of the importance of the findings and their relevance to future understanding of disease processes, diagnosis or treatment?

Partly

Is the background of the case’s history and progression described in sufficient detail?

Partly

Reviewer Expertise:

Internal Medicine, Infectious diseases, tuberculosis, chronic diseases, endocrinology, rheumatology, nephrology, hematology.

I confirm that I have read this submission and believe that I have an appropriate level of expertise to confirm that it is of an acceptable scientific standard, however I have significant reservations, as outlined above.

## References

[1] : Diagnostic accuracy of the PLASMIC score in patients with suspected thrombotic thrombocytopenic purpura: A systematic review and meta-analysis. *Transfusion* .2020;60(9) : 10.1111/trf.15954 2047-2057 10.1111/trf.15954 32757237

[2] : Role of the PLASMIC Score in the Management of Thrombotic Thrombocytopenic Purpura. *Cureus* .2023; 10.7759/cureus.36188 10.7759/cureus.36188 PMC1010442237065284

# Peer review: Reviewer response for version 2

**DOI:** 10.5256/f1000research.134308.r139003

**Published:** 2022-06-09

**Authors:** Ahmet Emre Eşkazan

**Affiliations:** 1 Division of Hematology, Department of Internal Medicine, Cerrahpaşa Faculty of Medicine, Istanbul University-Cerrahpaşa, İstanbul, Turkey

From my point of view, the authors have revised the paper successfully, so it can be accepted for indexing.

Are enough details provided of any physical examination and diagnostic tests, treatment given and outcomes?

Partly

Is the case presented with sufficient detail to be useful for other practitioners?

No

Is sufficient discussion included of the importance of the findings and their relevance to future understanding of disease processes, diagnosis or treatment?

Yes

Is the background of the case’s history and progression described in sufficient detail?

Partly

Reviewer Expertise:

TTP, Hematology

I confirm that I have read this submission and believe that I have an appropriate level of expertise to confirm that it is of an acceptable scientific standard.

# Peer review: Reviewer response for version 1

**DOI:** 10.5256/f1000research.54448.r100435

**Published:** 2022-01-11

**Authors:** Ahmet Emre Eşkazan

**Affiliations:** 1 Division of Hematology, Department of Internal Medicine, Cerrahpaşa Faculty of Medicine, Istanbul University-Cerrahpaşa, İstanbul, Turkey

I have reviewed the manuscript. Although relatively rare, SLE and TTP may occur in the same patient, and although the case was well-presented, I do not think that this case does add anything new to what we have already known on this subject. As the authors stated, there are no ADAMTS13 activity, antigen, and antibody results. This, especially the anti-ADAMTS13 antibody result, is really important to distinguish hereditary TTP from immune-mediated TTP. In addition, the direct antiglobulin test is also lacking. In the longer-term follow-up, the patient did not receive any treatment for SLE, which is quite interesting.

Are enough details provided of any physical examination and diagnostic tests, treatment given and outcomes?

Partly

Is the case presented with sufficient detail to be useful for other practitioners?

No

Is sufficient discussion included of the importance of the findings and their relevance to future understanding of disease processes, diagnosis or treatment?

Yes

Is the background of the case’s history and progression described in sufficient detail?

Partly

Reviewer Expertise:

TTP, Hematology

I confirm that I have read this submission and believe that I have an appropriate level of expertise to state that I do not consider it to be of an acceptable scientific standard, for reasons outlined above.

# Peer review: Reviewer response for version 1

**DOI:** 10.5256/f1000research.54448.r97915

**Published:** 2021-11-15

**Authors:** Katerina Pavenski

**Affiliations:** 1 Department of Medicine and Laboratory Medicine, St. Michael's Hospital - Unity Health Toronto, Toronto, Canada

This is a case report of a 32yo female with a new diagnosis of presumably immune TTP. The case is not unique but still may have value to the readers with appropriate revisions. I summarize my feedback below:

**Title**

 Based on reading this case report, I am not convinced that your patient fulfils the diagnostic criteria for SLE. She clearly has positive autoimmune serology but does not appear to have any clinical features of SLE. Finding of positive autoimmune serology is not uncommon in patients with iTTP, and some of these patients eventually develop SLE and some of them will not.

**Abstract**

1. Suggest to change "TTP often occurs as a complication of SLE" to "TTP sometimes is associated with SLE". The latter statement is more accurate. 
2. Re-word "direct Coombs negative hemolytic anemia". You surely do not mean "autoimmune" hemolytic anemia which is associated with spherocytes on film and RBC autoantibodies.
3. What is "conserved" renal function? Do you mean her renal function was at baseline on the basis of measuring her serum creatinine, or serum creatinine was in normal range? Was urinalysis done? 
4. Was troponin done to rule out cardiac involvement?
5. ADAMTS13 activity and inhibitor were not done in this case but are required to confirm diagnosis. Please discuss this limitation and way to mitigate it (i.e. in resource constrained circumstance, can use a prediction score - such as PLASMIC) to predict chance of having low ADAMTS13.

**Introduction**

1. The classical pentad occurs in under 10% of cases of TTP. It is therefore important to question TTP when there is MAHA, thrombocytopenia and no obvious explanation and not wait for the pentad.
2. Completely disagree with statement "generally, TTP is a complication in patients with SLE". In most patients, iTTP is not associated with SLE or any other disorder. Indeed, to see SLE and TTP is not common at all.

**Case presentation**

1. What is meant by mixed ancestry?
2. Does "irregular treatment" with carbamazapine mean inconsistent compliance?
3. Was there any history of obstetrical complications? Were antecedent births live and healthy? Were there any pregnancy losses?
4. Why were platelets transfused and repeatedly - 5 times? Based on lab work provided, very high chance of this being TTP (for e.g., if use PLASMIC score) and platelet transfusions are considered contra-indicated in patients with TTP (risk of worsening thrombosis) unless there is a life-threatening bleed. Was she seriously bleeding? 
5. Why was plasma exchange not started right away or only after worsening of clinical and labs? Based on presenting labs, very high index of suspicion that this was TTP...why PEX was stopped after 4 treatments? Standard of care is usually daily PEX until platelets are normal for at least 2 consecutive days. 

**Discussion**

1. I disagree with "TTP does not usually present alone". In fact, in majority of cases, TTP presents alone, with no other diseases. 
2. I understand that there was a diagnostic uncertainty about her initial diagnosis: SLE vs TTP. And that treatment of SLE was appropriately steroid pulse. However, based on her presenting picture (fragmentation hemolysis and thrombocytopenia and evidence of target organ damage - neurological) TTP could not be ruled out and as such (SLE or not) she should have received PEX or risk mortality or further organ damage. For every day delay in starting TPE, risk of death increases (Van de Louw *et al.,* 2021 ^1^). This should be the message of this case report.
3. I also would encourage the authors to include the statement about importance of getting ADAMTS13 activity test. If APLA, other autoimmune sophisticated serology could be done by the lab, so could be ADAMTS13. And this test is very important precisely because it is so difficult to differentiate TTP from other diseases, especially if more than one disease is present. 
4. Re sentence, "TTP should always be considered...however in young women SLE and TTP can occur simultaneously": This is not the reason to delay TTP treatment. TTP is most likely in young women and so is SLE. TTP should always be considered and treatment should be started, whether SLE is also suspected and especially in young women.

**Conclusion**

 Really, conclusion should be that SLE and TTP can occur together (albeit not frequently) and this makes diagnosis more difficult. However, considering the severe consequences of leaving TTP untreated (death, major organ damage), patient should be treated expediently as TTP with PEX and steroids while diagnosis is being sorted out.

 Please consider my suggestions and good luck.

Are enough details provided of any physical examination and diagnostic tests, treatment given and outcomes?

Yes

Is the case presented with sufficient detail to be useful for other practitioners?

Partly

Is sufficient discussion included of the importance of the findings and their relevance to future understanding of disease processes, diagnosis or treatment?

Partly

Is the background of the case’s history and progression described in sufficient detail?

Partly

Reviewer Expertise:

TTP and other thrombotic microangiopathies, therapeutic apheresis and trasnfusion medicine

I confirm that I have read this submission and believe that I have an appropriate level of expertise to confirm that it is of an acceptable scientific standard, however I have significant reservations, as outlined above.

## References

[1] : Outcomes in 1096 patients with severe thrombotic thrombocytopenic purpura before the Caplacizumab era. *PLoS One* .2021;16(8) : 10.1371/journal.pone.0256024 e0256024 10.1371/journal.pone.0256024 34383822PMC8360509

# Response

**Published:** 2022-05-21

**Authors:** Virgilio E Failoc-Rojas

**Affiliations:** Unidad de Investigación para la Generación y Síntesis de Evidencias en Salud, Universidad San Ignacio de Loyola, Lima, Perú, Peru

**Title**

 Based on reading this case report, I am not convinced that your patient fulfils the diagnostic criteria for SLE. She clearly has positive autoimmune serology but does not appear to have any clinical features of SLE. Finding of positive autoimmune serology is not uncommon in patients with iTTP, and some of these patients eventually develop SLE and some of them will not.

**Author Response:** In fact, the patient met the criteria for SLE, but we did not detail the clinical manifestations. The patient had a history of epileptic seizures and oral ulcer, and on admission presented with malar erythema. Given the initial suspicion of SLE, subsequent immunological tests confirmed the diagnosis (positive antinuclear antibodies in titers of 1/320, and positive antinuclear DNA in titers of 38.24 IU/mL). We have sought to clarify the diagnostic process of SLE in the text.

**Abstract**

 1. Suggest to change "TTP often occurs as a complication of SLE" to "TTP sometimes is associated with SLE". The latter statement is more accurate.

**Author Response: **Thank you. We have changed the statement.

 2. Re-word "direct Coombs negative hemolytic anemia". You surely do not mean "autoimmune" hemolytic anemia which is associated with spherocytes on film and RBC autoantibodies.

**Author Response: **We have re-worded the phrase.

 3. What is "conserved" renal function? Do you mean her renal function was at baseline on the basis of measuring her serum creatinine, or serum creatinine was in normal range? Was urinalysis done?

**Author Response:** We clarified this term. Yes, urinalysis was done and showed that serum creatinine was in the normal range.

 4. Was troponin done to rule out cardiac involvement?

**Author Response:** Yes, troponin was done and ruled out cardiac involvement.

 5. ADAMTS13 activity and inhibitor were not done in this case but are required to confirm diagnosis. Please discuss this limitation and way to mitigate it (i.e. in resource constrained circumstance, can use a prediction score - such as PLASMIC) to predict chance of having low ADAMTS13.

**Author Response: **Thank you, this limitation was discussed briefly.

**Introduction**

 1. The classical pentad occurs in under 10% of cases of TTP. It is therefore important to question TTP when there is MAHA, thrombocytopenia and no obvious explanation and not wait for the pentad.

**Author Response:** Thank you for your comment. We agreed with this idea; this was pointed out in the text.

 2. Completely disagree with statement "generally, TTP is a complication in patients with SLE". In most patients, iTTP is not associated with SLE or any other disorder. Indeed, to see SLE and TTP is not common at all.

**Author Response:** Thank you. We clarified this statement, as the message was to explain that TTP might occur after SLE as a complication, in the context of the rare presentation of both entities.

**Case presentation**

 1. What is meant by mixed ancestry?

**Author Response:** This was a term referred to as mestizo. We changed this term for sake of clarity.

 2. Does "irregular treatment" with carbamazapine mean inconsistent compliance?

**Author Response:** Yes, it does. To clarify the term, we changed it to “has been treated inconsistently with carbamazepine”.

 3. Was there any history of obstetrical complications? Were antecedent births live and healthy? Were there any pregnancy losses?

**Author Response: **The patient had three preterm delivery events. All births were live and healthy and there were no pregnancy losses. This was added in the text.

 4. Why were platelets transfused and repeatedly - 5 times? Based on lab work provided, very high chance of this being TTP (for e.g., if use PLASMIC score) and platelet transfusions are considered contra-indicated in patients with TTP (risk of worsening thrombosis) unless there is a life-threatening bleed. Was she seriously bleeding?

**Author Response: **Treatment was defined after consultation with experts based on clinical and platelet levels. We agree that this treatment is not the most appropriate but in the limited circumstances of the hospital it was the most accepted option.

 5. Why was plasma exchange not started right away or only after worsening of clinical and labs? Based on presenting labs, very high index of suspicion that this was TTP...why PEX was stopped after 4 treatments? Standard of care is usually daily PEX until platelets are normal for at least 2 consecutive days.

**Author Response:** The reason immediate treatment with plasma exchange was not performed was that TTP was not initially suspected, and the availability of treatment was not immediate to provide timely care. In addition, treatment with PEX was momentarily suspended after four treatments because, by expert consensus, the patient showed a favorable pattern in which the platelet level was increasing, and the DHL concentration was decreasing. Finally, upon seeing a consistent improvement, it was decided not to restart the plasma exchange. This response was also added to the text.

**Discussion**

 1. I disagree with "TTP does not usually present alone". In fact, in majority of cases, TTP presents alone, with no other diseases.

**Author Response:** Thank you for your comment. We agree with this statement and have therefore revised the term in the text.

 2. I understand that there was a diagnostic uncertainty about her initial diagnosis: SLE vs TTP. And that treatment of SLE was appropriately steroid pulse. However, based on her presenting picture (fragmentation hemolysis and thrombocytopenia and evidence of target organ damage - neurological) TTP could not be ruled out and as such (SLE or not) she should have received PEX or risk mortality or further organ damage. For every day delay in starting TPE, risk of death increases (Van de Louw *et al.,* 2021 ^1^ ). This should be the message of this case report.

**Author Response:** Thank you for this insightful comment. We recognize that this was a limitation and agree that PTT should have been appropriately ruled out regardless of the diagnosis of SLE. We have added this message in the text.

 3. I also would encourage the authors to include the statement about importance of getting ADAMTS13 activity test. If APLA, other autoimmune sophisticated serology could be done by the lab, so could be ADAMTS13. And this test is very important precisely because it is so difficult to differentiate TTP from other diseases, especially if more than one disease is present.

**Author Response: **Thank you for your kind suggestion. Indeed, ADAMTS13 activity testing is a critical step in the diagnosis of TTP. However, many hospitals in Peru lack these and other sophisticated tests due to cost. With this in mind, we have added a statement on the importance of ADAMTS13 so that it may benefit the proper clinical management of PTT in future events.

 4. Re sentence, "TTP should always be considered...however in young women SLE and TTP can occur simultaneously": This is not the reason to delay TTP treatment. TTP is most likely in young women and so is SLE. TTP should always be considered and treatment should be started, whether SLE is also suspected and especially in young women.

**Author Response:** Thank you. The sentence was modified. We agree that in young women with these characteristics, one should think about TTP regardless of concomitant disease, and in the face of this suspicion act in a timely manner.

**Conclusion**

 Really, conclusion should be that SLE and TTP can occur together (albeit not frequently) and this makes diagnosis more difficult. However, considering the severe consequences of leaving TTP untreated (death, major organ damage), patient should be treated expediently as TTP with PEX and steroids while diagnosis is being sorted out.

**Author Response: **Thank you for your suggestion. We have modified this section to convey a more precise conclusion.
